# Human melanoma brain metastases cell line MUG-Mel1, isolated clones and their detailed characterization

**DOI:** 10.1038/s41598-019-40570-1

**Published:** 2019-03-11

**Authors:** Ellen Heitzer, Arwin Groenewoud, Katharina Meditz, Birgit Lohberger, Bernadette Liegl-Atzwanger, Andreas Prokesch, Karl Kashofer, Diana Behrens, Johannes Haybaeck, Dagmar Kolb-Lenz, Harald Koefeler, Sabrina Riedl, Helmut Schaider, Carina Fischer, B. Ewa Snaar-Jagalska, Danielle de’Jong, Karoly Szuhai, Dagmar Zweytick, Beate Rinner

**Affiliations:** 1 Institute of Human Genetics, Diagnostic and Research Center for Molecular BioMedicine, Medical University of Graz, Graz, Austria; 2 Institute of Biology, Leiden University, Leiden, The Netherlands; 3 Department for Biomedical Research, Medical University of Graz, Graz, Austria; 4 Department of Orthopedics and Trauma, Medical University of Graz, Graz, Austria; 5 Institute of Pathology, Medical University of Graz, Graz, Austria; 6 Gottfried Schatz Research Center for Cell Signaling, Metabolism & Aging, Medical University of Graz, Graz, Austria; 7 BioTechMed-Graz, Graz, Austria; 8 EPO - Experimental Pharmacology and Oncology GmbH, Berlin, Germany; 9 Department of Pathology, Medical Faculty, Otto von Guericke University Magdeburg, Magdeburg, Germany; 10 Center for Medical Research, Medical University of Graz, Graz, Austria; 11 Institute of Molecular Biosciences, Biophysics Division, University of Graz, Graz, Austria; 12 Dermatology Research Centre, Translational Research Institute, School of Medicine, The University of Queensland, Brisbane, Queensland Australia; 13 Red Cross Transfusion Service for Upper Austria, Graz, Austria; 14 Department of Cell and Chemical Biology, LUMC, Leiden, The Netherlands

## Abstract

Melanoma is a leading cause of high mortality that frequently spreads to the brain and is associated with deterioration in quality and quantity of life. Treatment opportunities have been restricted until now and new therapy options are urgently required. Our focus was to reveal the potential heterogeneity of melanoma brain metastasis. We succeeded to establish a brain melanoma metastasis cell line, namely MUG-Mel1 and two resulting clones D5 and C8 by morphological variety, differences in lipidome, growth behavior, surface, and stem cell markers. Mutation analysis by next-generation sequencing, copy number profiling, and cytogenetics demonstrated the different genetic profile of MUG-Mel1 and clones. Tumorigenicity was unsuccessfully tested in various mouse systems and finally established in a zebra fish model. As innovative treatment option, with high potential to pass the blood-brain barrier a peptide isolated from lactoferricin was studied in potential toxicity. Brain metastases are a major clinical challenge, therefore the development of relevant *in vitro* and *in vivo* models derived from brain melanoma metastases provides valuable information about tumor biology and offers great potential to screen for new innovative therapies.

## Introduction

Melanoma brain metastases (MBM) are a serious complication of metastatic melanoma, with 50% of melanoma patients developing brain metastases during their disease^1–4^. Melanoma is one of the most aggressive and therapy-resistant human cancers with median survival of less than six months^2–7^. In recent years different and partly very promising therapy approaches against melanoma have evolved. Dabrafenib, vemurafenib and trametinib, kinase inhibitors, are mainly used for patients with BRAF V600E mutation. Monoclonal antibodies, such as pembrolizumab (anti-PD-1), ipilimumab (anti-CTLA4), and nivolumab (anti-PD-1), are successfully used to stimulate the immune system, while peginterferon alfa-2b, an anti-proliferative cytokine, often used as adjuvant therapy^8^. But still, there is a tremendous need to develop more effective therapies for the treatment of melanoma brain metastases^9^. To this end, new models to understand the biology of melanoma brain metastases are urgently needed.

Cancer in general and especially melanoma are characterized by their heterogeneous nature and various subpopulations within the tumor^10–16^. In most cases, only one subpopulation is targeted by a certain treatment while other cells are left unharmed and the surviving cells repopulate the tumor^17^. Thus, to address tumor heterogeneity therapeutically, combinations of therapies are needed in order to eliminate the bulk of the tumor and, at the same time, the critical subpopulations. Explanations for the phenomenon of tumor heterogeneity include different, but not necessarily mutually exclusive, theories including clonal evolution of cancer cells, the existence of cancer stem cells, and cancer cell plasticity. Tumors are thought to derive from tumor-initiating cells through diverse differentiation programs leading to one or more distinct subpopulations within a tumor. Melanoma-initiating cells (MICs) were shown to exhibit molecular and functional features similar to stem cells, which have unlimited self-renewal, the potential to initiate and maintain tumor growth and to differentiate into heterogeneous tumor cells^11,14,18–20^. Identification of MIC cells is not trivial; especially since no consensual marker characterizing the MICs population has been identified to date. Among other factors, such as CD34 and CD44, cancer stem cells (CSC) in melanoma or MICs express nerve growth factor receptor (NGFR), also known as CD271, on their cell surface^20^.

Several reports have shown that compared to CD271-negative cells, CD271-positive cells have a higher tumorigenic potential when injected into nude mice^10,18^. In contrast to the models of cancer stem cells and clonal evolution, phenotypic plasticity stands as an independent source of heterogeneity. The major part of phenotypic heterogeneity in melanoma is therefore not associated with a loss of tumorigenic potential or organized in stable hierarchies^21^.

The molecular mechanisms underlying the phenotypic heterogeneity are very complex showing genetic, epigenetic and environmental components, such as shortage in oxygen or energy supply by triacylglycerides. Often, features other than specific surface markers, i.e. different tumor potential and aggressiveness, are commonly used to define subpopulations within a tumor or a cancer cell line.

Here we aimed to (i) demonstrate the heterogeneity and presence of subpopulations of melanoma brain metastasis and to (ii) develop a relevant *in vitro* and *in vivo* model, which can be used for the development of more effective therapies. We succeeded to establish a brain melanoma metastasis cell line (MUG-Mel1). To be noticed, that wildtype BRAF, NRAS and cKIT angle genetic background is less common among currently established cell lines and makes our established cell line special. To elucidate the heterogeneity of the cell line, we isolated two subclones, C8 and D5, based on single cell sorting. In order to characterize the heterogeneity of the different cell lines in more detail we performed a variety of experiments including tumorigenic potential, proliferation and migration assays, stemness characteristics, karyotyping, electron microscopy and lipidom. In a previous work we showed that the negatively charged phospholipid phosphatidylserine (PS) is highly exposed on the outer leaflet of the plasma membrane of tumor cells, which is a target for PS-specific host defense derived peptides for novel therapeutic approaches^22^. Therefore, we additionally investigated the treatment effect of R-DIM-P-LF11-322, an antitumor peptide, in our cell lines, which might represent a novel therapeutic approach for chemo-resistant cell types.

## Results

### Establishment of MUG-Mel1 and clones C8 and D5

Tumor material for the establishment of MUG-Mel1 cell line was retrieved from a biopsy of a brain metastasis of a 61 year old male patient. The patient was diagnosed with two primary, BRAF-negative, stage IV melanomas. Inguinal sentinel node biopsy revealed lymph nodes without any melanoma formations, whereas axillar node fatty tissue revealed melanoma formation in six out of ten lymph-nodes. The patient received an alpha interferon therapy, resulting that the cell line was not established from a treatment-naïve patient. MRI imaging revealed cerebral tumor lesions in the left temporal region, in the right parietal region and right occipital region. Biopsies revealed a metastatic malignant melanoma. Later, liver and lung metastases were also diagnosed. The study was approved by the local ethics committee board of the Medical University of Graz (vote #26-219ex13/14; valid until 22.12.2018), the patient gave written informed consent for the study specific procedure and was anonymized. All experiments were performed in accordance with relevant guidelines and regulations.

The primary melanoma tissue revealed typical features of a melanoma including expression of S100, HMB45, and Melan A. Likewise, the metastatic brain tissue clearly showed melanoma-specific markers. However, while almost all cells are S100 positive, only a part of cells stained positive for HMB45 and Melan A (Suppl. Fig. 1A–D). Interestingly, HMB45 and Melanin A were not expressed in the MUG-Mel1 cell line. Nevertheless, HE staining of the MUG-Mel1 cell line showed typical morphology of a melanoma cell line with prominent S100 positive cells (Suppl. Fig. 1E–G). Phenotypic characteristics of MUG-Mel1 cell line were manifold and presented a variety of cells including fusiform, round and flat or epithelial-like cells, with partially prominent cell nuclei and cells with long foothills (Suppl. Fig. 1E–G).

To further investigate the heterogeneity of the cell line we isolated single cell clones. Sorting of earlier passages of MUG-Mel1 (4, 13, 14) did not result in any expansion of the cells. However, after sorting of passage 55 into five 96 well plates under the same conditions a total of 10 clones started growing. Finally, we were able to expand two completely morphologically different clones called clone MUG-Mel1 C8 (C8) and MUG-Mel1 D5 (D5). While clone C8 showed a spindle shaped morphology in culture, clone D5 presented round and flat growth behaviour. During cultivation and passaging minor morphological changes were observed, in particular clone D5 showed morphological changes from round shaped cells to fusiform cells. However, the typical morphological features of each clone have been maintained since both clones were grown for six month to passage 100 to ensure continuous growth. To prove the common origin of the tumor samples, STR profiling of the parental cell line and the two subclones was performed. All analyzed samples showed the same tandem repeat (STR) profile at the markers CSF1PO, D3S1358, D18S51, D8S1179, D5S818, D13S317, D7S820, D16S539, Penta D, vWA, TH01, TPOX, FGA and Amelogenin confirming that MUG-Mel1, C8, and D5 emanated from the same patient (Suppl. Table 1).

Based on mutation analysis using the AmpliSeq HotSpot panel MUG-Mel1 did not harbour any mutation in the most recurrently mutated genes in melanoma such as in a *BRAF*, *NRAS*, *KIT*. However, *NF1* mutation (NM 001042492: c.A5233G, p.K1745E) was identified, which could also be detected in the primary tumor, MUG-Mel1 cell line and the two subclones C8 and D5. Furthermore, western blot revealed p53 deficiency (data not shown).

Molecular karyotyping revealed a high level of aneuploidy, which is a remarkably common feature of human cancer, present in ∼90% of solid human tumours. To assess the copy number status of the cell line more accurately and to compare the copy number alterations (CNAs) of MUG-Mel1 and its subclones to the respective tumor tissues, we performed low coverage whole genome sequencing and Affymetrix 6.0 SNP Arrays.

**Figure 1:**
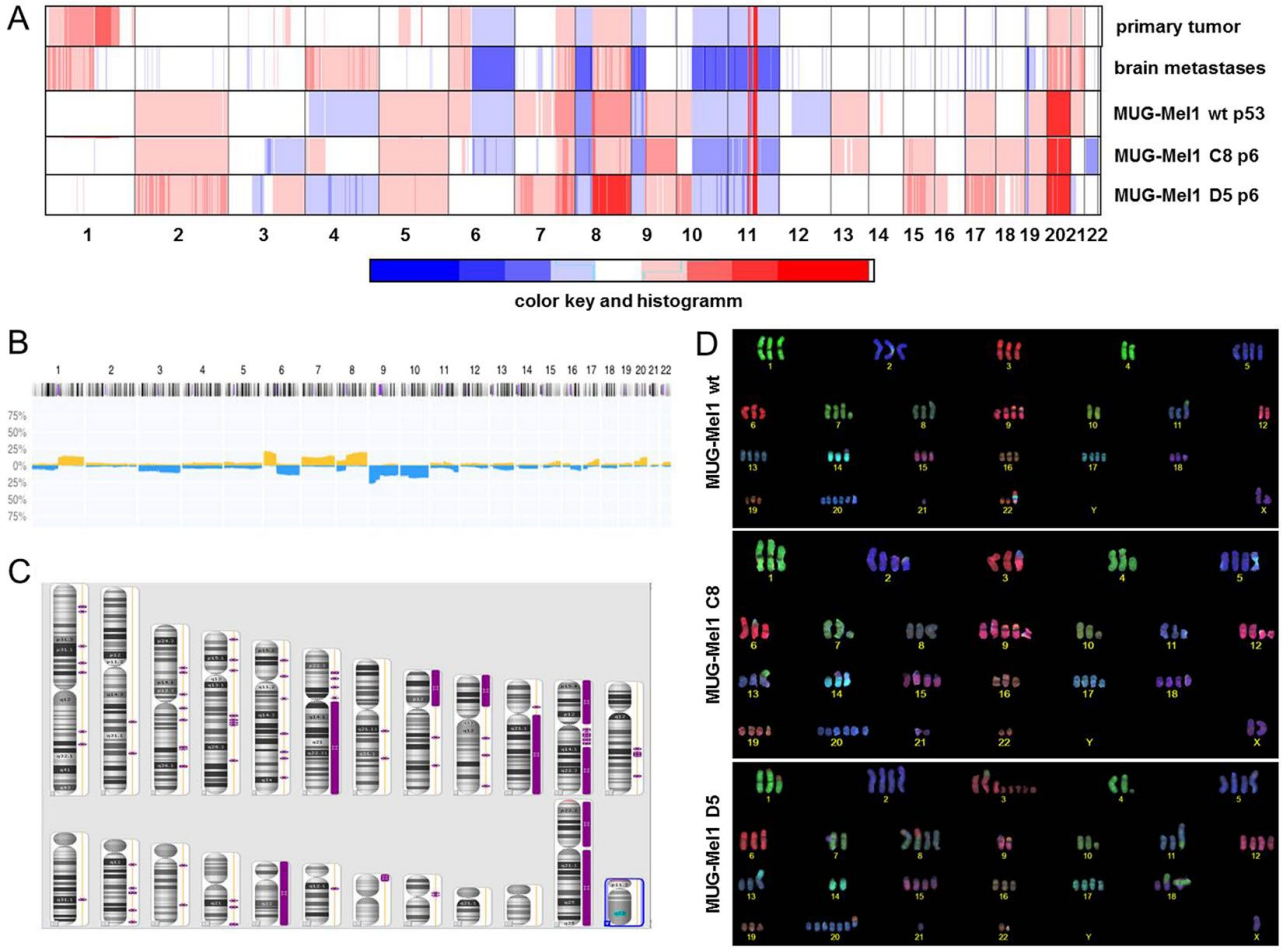
Copy number profile. Copy number profiles of the primary melanoma tumor tissue, brain melanoma tumor tissue, and the newly established cell line MUG-Mel1 passage 53 and resulting clones C8, passage 6 and D5, passage 6 were done. Depicted are log2 ratio plots of the genome. Regions with log2 ratios >0.2 that indicate gains of chromosomal material are shown in red and those with log2 ratios <−0.2 that indicate loss of chromosomal material are shown in blue. Balanced genomic regions are depicted in green. (**A**) Publicly available copy number data from the Progenetix database. Plotted is the frequency (x-axis) of gains (yellow) and losses (blue) across the genome of 230 samples with ICD morphology code 8720/3: Malignant melanoma, NOS (M-87203). Publically available copy number data from Progenetix database. (**B**) Loss of heterozygosity presented from MUG-Mel1 (**C**); COBRA-FISH analyses MUG-Mel1, clone C8 and clone D5 (**D**).

The primary melanoma and the metastatic brain tissue showed a high degree of similarity clearly demonstrating the common origin. A variety of CNAs i.e. gains on chromosome 1p, 6p, 7q, 8q, 20 and 21 as well as losses on chromosome 6q, 8p, 9p, 10q, 11 and 23 (Fig. 1A) were highly concordant with available copy number data for melanoma from the public Progenetix database (Fig. 1B). Some of these regions additionally involved loss of heterozygosity (LOH) (Fig. 1C). Most of the CNAs from the tumor tissues were also observed in MUG-Mel1, although the overall number of CNAs was higher in the cell line compared to the respective tumor samples in particular affecting the small chromosomes 13, 17 and 19. Moreover, the cell line showed gains of chromosomes 2 and 5. Whether these putative *de novo* CNAs represent cell line artifacts that arose during passaging of cell cultures or represent potential somatic mosaicisms that were cloned during the establishment of the cell culture is not clear yet. Interestingly, although the majority of genomic regions showed identical CNA in MUG-Mel1 and the two single cell clones C8 and D5, some regions were dissimilar indicating that substantial chromosomal alterations might contribute to the different phenotypes. The most prevalent differences included chromosomes 6, 7, 13, 22, 3q, 4p, and 10p.

### Karyotyping experiments

COBRA FISH karyotyping revealed a complex, near triploid karyotype, with a consistent loss of the Y chromosome and stably occurring chromosomal alterations in all three analyzed cell lines. All these finding were in line with the moderate gain-loss and the high gain pattern observed with array-CGH when samples with a 3n ploidy are normalized.

**Figure 2:**
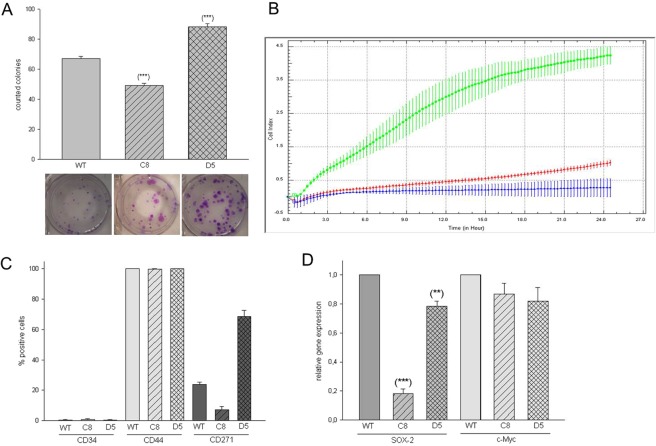
Surface and Stemness Marker Characterization. Presentation of stem cell characteristics: CFU assay, D5 present significantly more CFU compared to MUG-Mel1 and C8, representative pictures of three biological replicates (**A**); Migration behavior detected by xCELLigence, D5 (line green) presented a high migration potential compared to C8 (line red) and MUG-Mel1 (line blue) representative pictures of three biological replicates (**B**); Flow cytometry measurements of CD34, CD44, and CD271, D5 present the highest amount of CD271 positive cells and was measured in triplicates (**C**); detection of various stemness marker, D5 compared to C8 expresses a significant higher level of SOX-2, whereas no significant difference regarding c-Myc was detected (n = 3) (**D**).

In the MUG-Mel1 cell line stably occurring structural chromosomal alteration included isochromosome 6p, iso(8q), der(9;22)t(q10;q10), isoder(11q)t(7;11)t(11;21), rob(14;21) and random non-clonal rearrangements. The metaphase cells showed four copies of chromosome 2, 5, 7, 13, 17 and 19, six or more copies of chromosome 20 and two copies of chromosome 4. In the MUG-MEL1-C8 cell line several stably occurring chromosomal alteration were detected, including an isochromosome 6p, iso(8q), der(9;22)t(q10;q10), ider(11q)t(7;11)t(11;21), rob(14;21) and random non-clonal rearrangements. These cells showed four copies of chromosome 2, 5, 7, 13, 17 and 19, 6 or more copies of chromosome 20. Translocation involving chromosome 3 and an additional copy of chromosome 15 were in line with the loss of the chromosome 3 and gain of chromosome 15 detected by array-CGH, respectively (Fig. 2D). In the D5 cell line next to several stably occurring chromosomal alterations a change in the overall ploidy from near triploid to hypertriploid and hypotetraploid was observed (72–83 chromosomes in different metaphase cells). An extra copy of isochromosome 8q and loss of the isochromosome 6p and derivative chromosome 3q fragments could explain the changes observed with array-CGH, namely the gain of chromosome 3q regions, the “normalization of chromosome 6” and the high gain of chromosome 8q (Fig. 1D).

### Assessment of Tumorigenicity

The *in vitro* tumorigenic potential of the cell line was investigated by soft agar assay for colony formation to determine the ability of one single cell to grow into a large colony. After an incubation time of 30 days an average of 67.08 +/− 5.07 MUG-Mel1 colonies per well were counted, 49.33 +/− 4.98 C8 and 88.33 +/− 7.09 D5 (representative example Fig. 2A) confirming cellular anchorage-independent growth. Significant difference in forming colonies between MUG-Mel1, C8, and D5 was investigated.

Cell growth was determined by doubling time and measured by xCELLigence real time system. Surprisingly, the parental MUG-Mel1 grew faster than D5 and C8 cells with a doubling time of 32.16 ± 4.28 hours compared to 50.62 ± 8.63 and 75.18 ± 20.11 hours, respectively for C8 and D5 (data not shown). The effects of migratory properties were determined using uncoated CIM-16 xCELLigence plates in three biological replicates. While MUG-Mel1 and C8 exhibited no migration potential, D5 possessed the ability to migrate from serum free medium to media with serum within 24 h (Representative illustration of triplicates Fig. 2B). Identification of stemness markers was done by flow cytometry and RNA analyses. Expression of surface marker CD271, CD34 and CD44 was measured by flow cytometry in triplicates. All three cell types represented CD34 negativity but high CD44 expression, although levels of fluorescent intensity were slightly different between the different populations (Fig. 2C). The highest mean fluorescent intensity of CD44 was detected in MUG-Mel1 (36.647), followed by D5 cells (24.591) and C8 (21.675). D5 presented a significantly higher expression of CD271 compared to MUG-Mel1 and C8. Additionally, using quantitative PCR the expression of SOX-2 and c-Myc was investigated. Both subclones showed a significantly decreased expression of SOX-2 with reductions to 20% and 80% for C8 and D5 compared to the parental cell line. In contrast, no significant differences were observed for the expression of c-Myc (Fig. 2D).

### Electron microscopy

**Figure 3:**
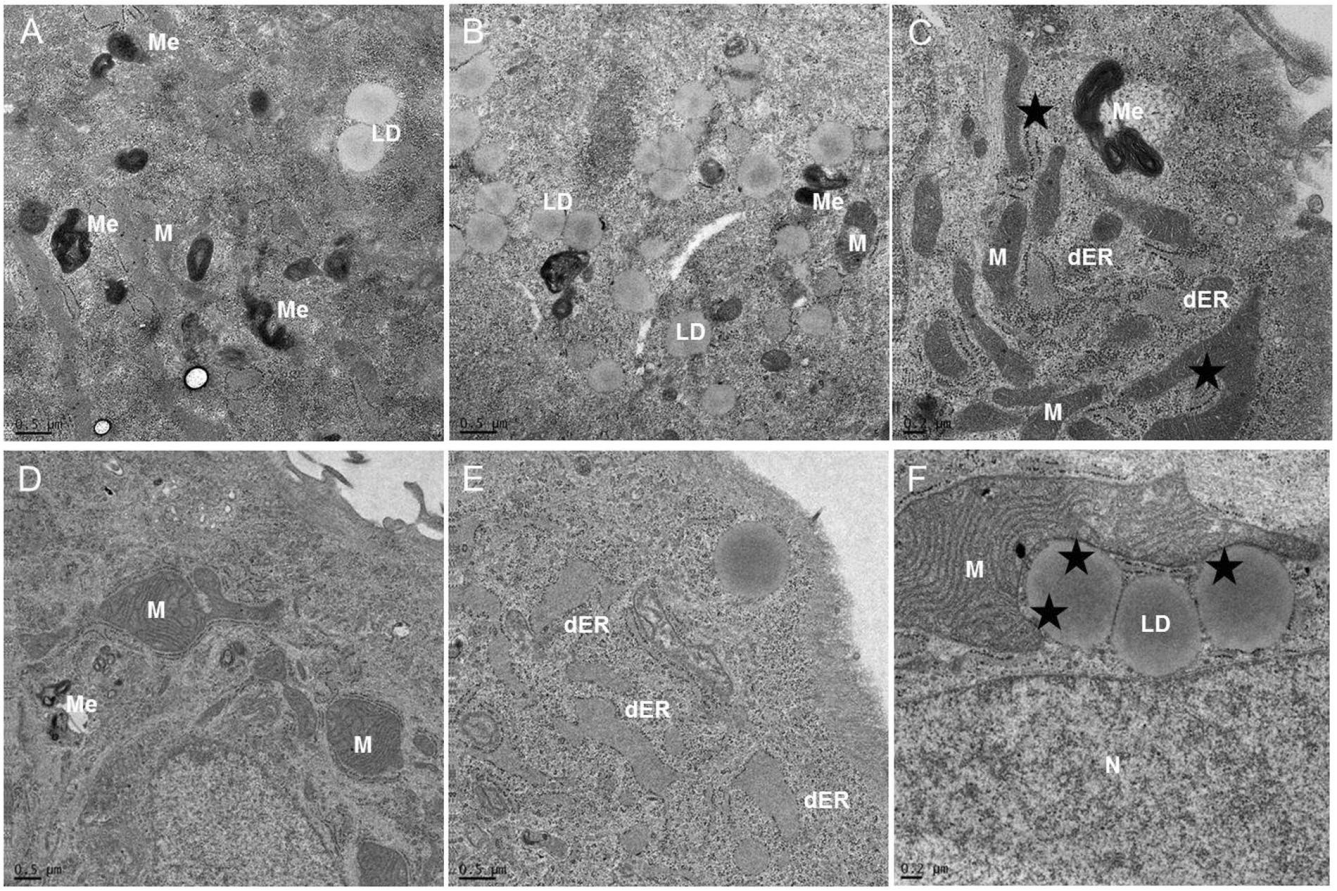
Electron Microscopy. Electron micrographs indicate the presence of mitochondria, lipid droplets and melanosoms. MUG-Mel1 clone C8 showed a high number of elongated mitochondria (M), various melanosomes (ME), lipid droplets (LD) (**A**–**C**); MUG-Mel1 clone D5 showed enlarged mitochondria, melanosoms, dilated rough endoplasmic reticulum (dER) and large mitochondria in close connection with lipid droplets (LD), Nucleus (N) (**D–F**).

Since histologic heterogeneity of tumors is a well-known phenomenon, and in order to confirm the melanocytic differentiation with melanosomes as an ultrastructural marker, we analyzed the cells by electron microscopy. Thereby we observed morphologic differences regarding mitochondria, endoplasmatic reticulum (ER), lipid droplets, and lysosomes. D5 and C8 showed a high number of elongated mitochondria, various melanosomes, lipid droplets, mitochondria and ER presented in close connection and partly dilated ER. Main difference was detected in the form of mitochondria and connection with ER (Fig. 3A–C: C8; D–F: D5). As expected the parental cell line MUG-Mel1 presented both morphological phenotypes (Suppl. Fig. 2).

### Lipid Analysis

**Figure 4:**
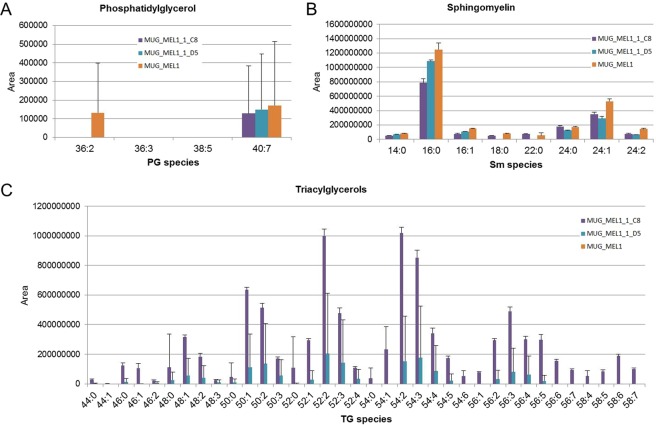
Lipid Profiles. Lipid profiles of phosphatidyl glycerol (**A**), sphingomyelin (**B**), and triacylglycerol species (**C**) in cell lines MUG-Mel1, C8, and D5 (n = 4 each) determined by LC-MS/MS.

Based on the electron microscopy data we intended to emphasize the heterogeneity of C8 and D5 and performed a non-targeted differential lipidomic analysis, since the number of lipid droplets appeared to be different. In this assay the level of Triglyceride (TG) was strongly upregulated in C8 cells (Fig. 4). Particularly, TG species containing very-long-chain polyunsaturated fatty acids (VLC-PUFA) - FA20:3, 20:4, 20:5, 22:4, 22:5, 22:6, and 24:6 - were detected only in C8 cells (Fig. 4C).

**Figure 5:**
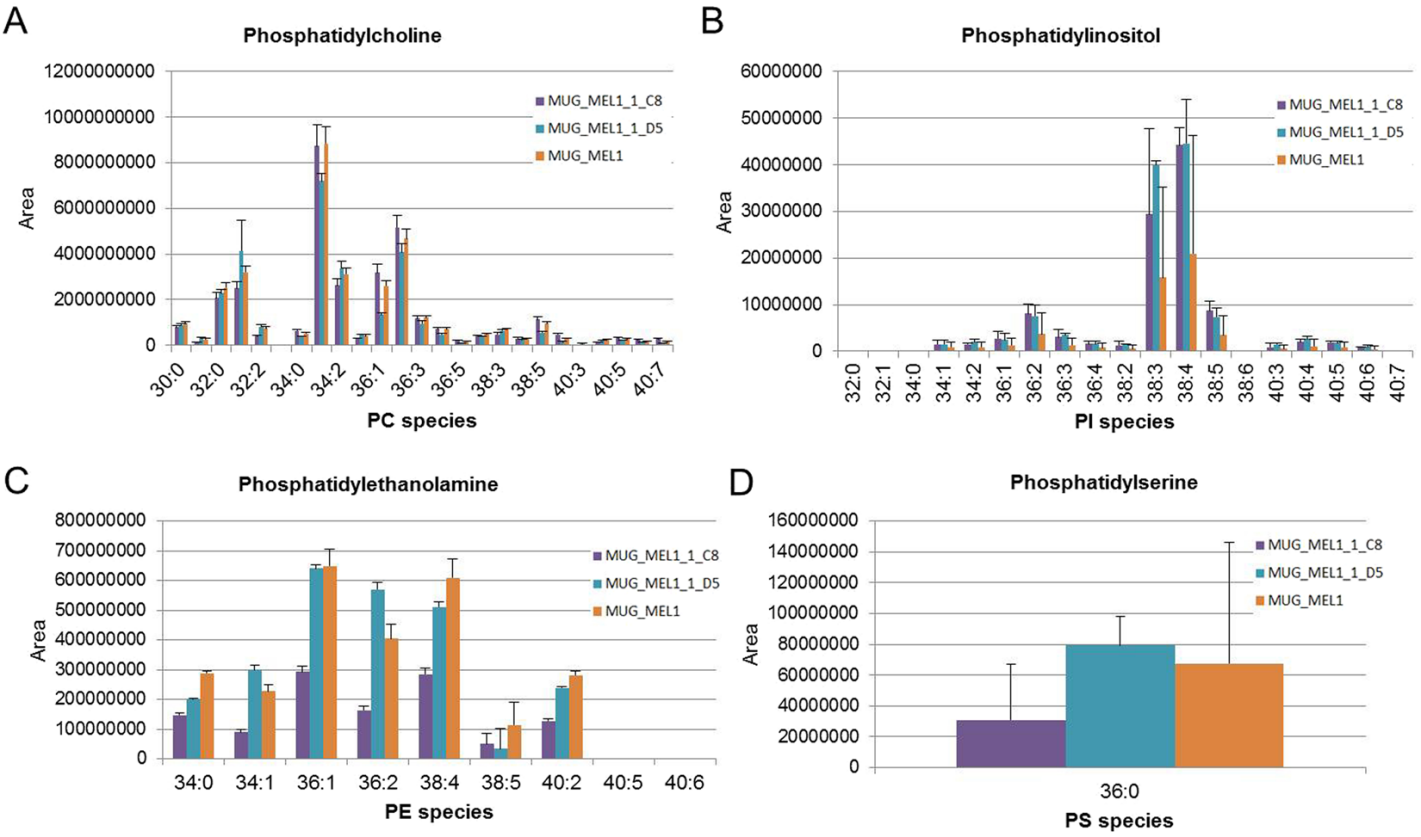
Lipid Profiles. Lipid profiles of phosphatidyl choline (**A**), phosphatidyl inositol (**B**), phosphatidyl ethanolamine (**C**) and phosphatidyl serine (**D**) in cell lines MUG-Mel1, C8, and D5 (n = 4 each) determined by LC-MS/MS.

Phospholipids (PL) showed in comparison to D5 cells a slight but not significant trend for upregulation in C8 cells, particularly PC 40:5, PC 40:6, and PC 40:7 while e.g. PC 34:2, PC 32:1, and PC 32:1 were downregulated compared to D5 (Fig. 5A). Phosphatidyl inositol (PI) species, on the contrary, showed a trend towards up regulation in C8 and D5 cell lines compared to MEL1 cell lines (Fig. 5B). Some PL classes like phosphatidyl ethanolamine (PE) and phosphatidyl serine (PS) were down regulated in C8 cells (Fig. 5C,D).

### ***In vivo*** models

**Figure 6:**
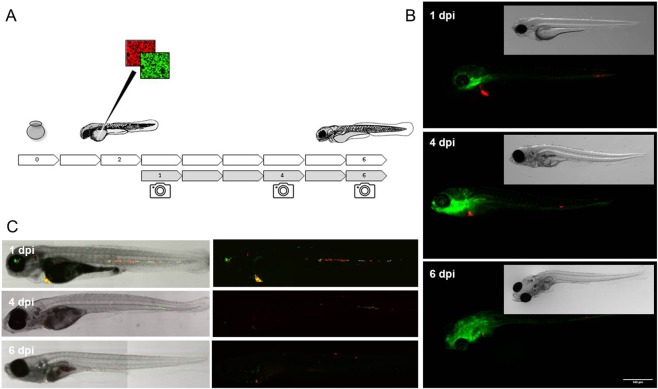
Zebra Fish. Schematic overview of the experimental approach, embryos are collected at day 0 and injected at 2 days post fertilization (dpf) through the embryonic common cardinal vein (Duct of Cuvier), following the embryos are imaged on 1, 4 and 6 days post injection (dpi) to follow the initial dispersal of the cells at 1 dpi, the stage of engraftment around 4 dpi and eventual successful outgrowth of micro metastatic foci at 6 dpi. (**A**) Fluorescent micrographic images of fli:GFP blood and lymphatic vessel reporter zebrafish engrafted with MUG-Mel1 (red), imaged on day 1, 4 and 6 dpf show dispersal, engraftment and outgrowth of experimental micro metastases. (**B**) Confocal stich of ABTL zebrafish embryos engrafted with 1:1 mixed clones, C8 (green) and D5 (red), after injection clone C8 clearly does not form any multicellular foci whereas D5 establishes multiple foci 6 days after engraftment. (**C**) All images represent median phenotypes of injected cohorts, scale bars represent 200 µm.

In order to test the tumorigenic potential of the cell lines *in vivo*, the parental CD271 FACS sorted MUG-Mel1 as well as subclones C8 and D5 were injected with 1:1 matrigel and w/o matrigel into mice models including nude mice, NOD-SCID mice and NSG mice. Xenotransplantation under the skin as well as organotrophe models in mouse brain were used. However, after 2 month no tumor mass was detectable. Even after scarificing the mice and careful investigation of harvested organs and tissues no tumors or micrometastases were detected. Since engraftment of all cell lines did not lead to any observed morbidity upon comparison with uninjected siblings. The maternal cell line MUG-Mel1 was capable of generating small micro metastatic foci as defined in Fig. 6, although the majority of the cells did not yield viable foci some pervasive foci were present up to the moment of termination. Both maternal line MUG-Mel1 and the derived clones C8 and D5 were capable of forming micro metastatic foci after intravenous injection into zebrafish, remarkably D5 proved more tumorigenic when compared to C8 with only single cells remaining for clone C8 and obvious multicellular foci for D5. Engraftment of an equal ratio of both C8 and D5 clearly indicates a heightened tumorigenic potential for clone D5 in the zebrafish model.

### Peptide treatment

**Figure 7:**
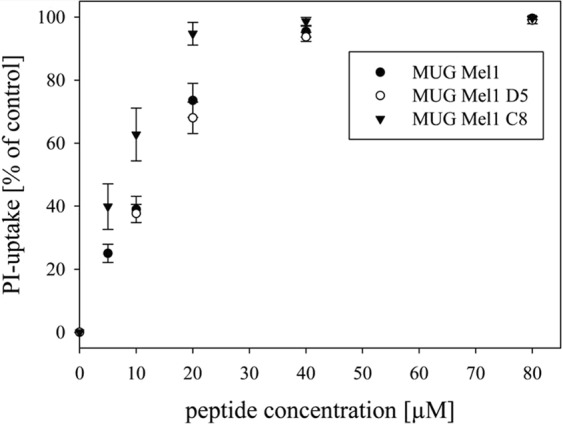
Peptide Treatment. The lines present cell death induced by increasing peptide concentrations determined by % PI-uptake of cell lines MUG-Mel1, D5, and C8 after 8 h of incubation. LC_50_ values are demonstrated in Table 1.

Membrane-active antitumor peptides that target a lipid specifically exposed by cancer cells and metastases were studied in their toxicity on the maternal line MUG-Mel1 and its clones due to the fact that they act independent on cancer (cell) type or mutations and are even prone to pass the blood brain barrier due to their small size and lipophilic character. Cytotoxic activity of the peptide R-DIM-P-LF11-322 towards MUG-Mel1, D5, and C8 was determined by measurement of PI-uptake, which is an indication for the loss of cell membrane integrity and cell death (Fig. 7). R-DIM-P-LF11-322 showed similar activities for MUG-Mel1, D5, and C8 comparable to A375 control cells^21^. R-DIM-P-LF11-322 exhibits high activity against all cancer cell lines but yielding more than 50% killing after 4–8 h of incubation with a preference for C8 (94.7% killing at 20 µM peptide concentration after 8 h). MUG-Mel1 and D5 show slightly reduced PI-uptake of 73.6% and 68.1%, respectively. The A slow killing of by R-DIM-P-LF11-322 within 4–8 h was observed (data for one, two, and four hours not shown) is in accordance with previously shown data reports indicating cell death through induction of apoptosis^23^. LC_50,_ 8 h values are displayed in Table 1. Again the peptide displays the highest activity on C8 cells by showing the lowest LC_50_ value (6.6 µM). MUG-Mel1 and D5 reveal marginally higher LC_50_ values of 11.3 µM and 10.6 µM, respectively, consistent with A375 cells (9.5 µM).

**Table 1:** Comparison of % killing at 20 µM and IC_50_ values of R-DIM-P-LF11-322 treated MUG-Mel1, D5, C8, and A375, determined by PI-uptake after 8 h. Data for PI measurements from at least five experiments are presented as mean ± SD.

cell line clone Peptide	MUG-Mel1	MUG-Mel1 D5	MUG-Mel1 C8	A375*
% killing 20 µM	73.6 ± 1.0	68.1 ± 5.0	94.7 ± 3.6	96.3 ± 3.6
LC50_8h_	11.3 ± 1.0	10.6 ± 1.9	6.6 ± 0.6	9.5 ± 0.3

*Riedl *et al*. (BBA 1848 (2015) 2918–2931).

## Discussion

Despite several treatment options including surgery or systemic therapies, the prognosis for overall survival of patients with brain metastasis remains poor^24^. Especially decisions on individualized therapies in patients with brain metastases are often made based on primary tumor material. Brastianos *et al*., proclaimed that the existing genetic divergence between clinically studied primary tumors and brain metastases cannot be detected by the analysis of a single sample of the primary tumor, biopsies from the primary tumor alone can miss a considerable number of opportunities for target therapy^25^.

In order to predict clinical response, to help generate pharmacogenomics hypotheses for further testing, and to help identify novel mechanisms associated with variations in drug response, human cell line models have been widely used. Cell lines are well matched to the cancer of interest and mirror tumor genetics and phenotype as closely as possible. Here we report the establishment and detailed characterization of a cell line derived from a melanoma brain metastasis. Although melanoma brain metastases are very common and contribute to 20–50% of melanoma related deaths^26^, only a few melanoma brain metastases cell lines for *in vitro* studies are available. Krepler *et al*., collected brain metastases tissues to generate PDX models. Mutation analyses from 16 samples were done and showed one BRAF/RAS, nine BRAF, six RAS mutations, and two BRAF, RAS wildtypes. Several cell lines were derived from the obtained samples. However, none of these depicted the combination of BRAF/RAS/cKIT wildtype^27^. Additionally BRAF/RAS/cKIT wildtype cell lines are currently not purchasable. Therefore MUG-Mel1, bearing this wildtype combination has a unique value for the melanoma research community. The establishment of MBM cell lines turned out to be difficult, as the preservation of phenotype in melanoma cell lines is quite poor and strongly depending on tissue culture conditions^28^. For example, absence of HMB45 staining as well as loss of Melan A, two typical markers of melanoma, is a common phenomenon in cell culture^29^. The only constantly present diagnostic marker under varying cultivation conditions is the tumor maker S100 protein^24^. This is consistent with our observation that MUG-Mel1 presented a S100 positive staining while Melan A and HMB45 were absent. In addition to culture conditions, a possible explanation might be heterogeneity of MBM, which showed differentially stained areas of HMB45, Melan A, and S100. Yet a study from Wang *et al*. contradicts this theory. The authors observed loss of Melan A in brain tumor melanoma tissue compared to primary skin tumor. When cultured spheroids from the MBM were injected into rat brains, Melan A expression could be restored^30^.

In addition to the loss of reactivity to conventional melanoma recognizing markers, we observed several other interesting findings during the establishment of these cell lines.

MUG-Mel1 presented two cell types with clearly different features regarding morphology and lipid droplets. Electron microscopy analysis allow a real-time observation of wildtype cell line and clones, quantification proves to be extremely difficult and must be explored in future experiments. To study the heterogeneity in the cell line, we employed single cell sorting to separate the two morphologically distinct cell lines. Since the sorting process is a challenge for survival of freshly isolated primary cells, sorting of earlier passages did not result in any cell expansion. However, cells of later passages with cell bounds and continuous proliferation rate tolerated cell sorting and two expanded single cell clones could be generated. Therefore, it is of note that not all primary cells from the tissue are reflected in the cell line since it is most likely that only the stable, robust cells conferring the dominant clones have survived.

In addition to phenotypical differences the two subclones showed differences of copy number alterations affecting chromosomes. Variations of the copy number status were detected at chromosomes 3q, 4, 6q, 7, 10p, 13, 16, and 22. Discrimination between benign from malignant melanocytic lesions can also be done by utilizing fluorescence *in situ* hybridization (FISH), whereas especially chromosome 6 (RREB1, MYB and Cep6) is mentioned as melanoma biomarker^31,32^. Cobra FISH analysis of MUG-MEl1 and clones also revealed aberration on chromosome 6.

Since melanoma cells show phenotypic features of melanocytes, which are derivatives of the neural crest stem cells and the current understanding of tumorigenesis and metastasis in melanoma is founded on the concept of cancer stem cell theory, we checked whether the subclones C8 and D5 show differential expression of the stemness marker CD271 and SOX-2.

Santini *et al*. found that SOX-2 expression is enhanced in melanoma cells with stem cell features^33^ and the neural crest stem cell marker CD271 expression was shown to be specifically increased in metastatic melanoma to the brain^34^. Interestingly, the round footed cells of subclone D5 showed higher expression of CD271 and SOX-2 compared to subclone C8 and the parental cell line MUG-Mel1. This was in line with data of the CFU assay, where D5 cells showed a significant increase in colonies, which clearly emphasize the stem cell-like character. Nevertheless, it is important to mention, that we were unable to prove *in vivo* tumorigenic effects in both mice and organotrophic models. Even using sorted CD271+ cells from D5, we did not observe tumor growth. Similar data were reported by Cheli *et al*., who distinguished between fast growing CD271+ without tumorigenic potential and slow growing CD271+ with the ability to form tumors in mice^35^, indicating that CD271 is an imperfect marker of melanoma initiating cells. Phenotypic heterogeneity in melanoma patients is largely driven by reversible changes in a broad range of markers that turn on and off, contrasting with both cancer stem cell and clonal evolution models^21^. The phenotypic heterogeneity in melanoma might explain that the clones and the CD271+/− cells show no *in vivo* grow. Since there are hardly any publications on BRAF and NRAS wild type melanoma brain metastases cell lines, there are no results regarding the xenotransplantation behavior of this type of cells.

Using Zebra fish model clone D5 proved more tumorigenic and indicates increased tumorigenic potential, which is consistent with the above mentioned stem-cell like features in this subclone. Since metabolic dysregulation is now firmly established as a hallmark of cancer^36^ we intended to assess fatty acid (FA) uptake and consumption in the two distinct subclones. Recently, Kwan *et al*. showed that lipids were transported from the adipocyte to melanoma in a co-culture system, whereas the melanoma cells enhanced proliferation compared to non-culture growth^37^. The unique feature of adipocytes is to store triacylglycerols and to release fatty acids for other organs and tissues^38^. Since most cancers have altered metabolic activity, the development of a lipogenic phenotype plays an important role in cancer^37^. Fatty acid synthesis is activated in various cancer types and inhibition of fatty acid synthases induces apoptosis^37,39^. Triacylglycerols provide a reservoir of fatty acids that can be mobilized for the generation of energy. Therefore, compared to normal tissue cancer cells seem to contain increased numbers of lipid droplets for storage of triacylglycerols and cholesteryl esters. To this end, mainly short chain fatty acids such as 2:0; 4:0 and 6:0 TG are used as energy source, whereas an increasing amount of saturated phospholipids (PLs) markedly alter signal transduction^40^. We showed that TGs were strongly upregulated in C8 cells, suggesting that C8 cells might be transmitting energy within the tumor. The most significant differences were found in triacylglycerols containing VLC-PUFAs, indicating the availability of precursors for oxygenated signaling lipids like eicosanoids and resolvins. In contrast to TGs, PE species were downregulated in both C8 and D5 cells, whereas PI species were upregulated. Since PI species are highly involved in transmembrane signal transduction processes in membrane domains like caveolae, this too indicates a high signaling and/or metabolic activity of the C8 subclone. Yet, the two subclones showed marked differences in both the size and amount of lipid droplets and the lipid profiles. The lipid profiles of the three cell lines suggest that the C8 subclone is the metabolically most active one whereas the D5 subclone basically shows the same patterns but in a less pronounced manner. Nevertheless one has to keep in mind that lipid metabolism is not a static affair and further stable isotope labeled studies are needed for a more dynamic picture which also reflects the functions associated with lipid certain patterns.

We recently reported the apoptotic effect of human lactoferricin derived di-peptides on cancer cells through targeting membranous phosphatidylserine^22^. Di-peptides are very small, lipophilic molecules; therefore it is likely that these molecules are able to pass the blood-brain barrier. Since the peptides have already been shown to exert specific antitumor activity on many cancer types, including melanoma and their metastases^22,23^ we treated MUG-Mel1 with different concentrations of lactoferricin derived R-DIM-P-LF11-322. The peptide induced cell death of MUG-Mel1, C8, and D5 with an IC_50_ of 11.3, 6.6 and 10.6 µM, respectively similar to values obtained for the melanoma cell line A375^23^. Importantly clone D5 expressing high levels of the stem cell marker CD271 was as well sensitive to the peptide as the maternal cell line. Further the peptide has already been shown to be highly selective for cancer cells with negligible toxicity on non-neoplastic cells as melanocytes or dermal fibroblasts^23^.

Taken together, we present the establishment and comprehensive characterization of a new melanoma brain metastases cell line and two associated subclones at multiple levels. We performed a variety of experiments including tumorigenic potential, proliferation and migration assays, stemness characteristics, karyotyping, electron microscopy, and lipidome, which clearly demonstrate the complexity and heterogeneity of brain metastases. Our data highlight the need for a better understanding of the underlying mechanism to improved treatment strategies and research directions.

## Material and Methods

### Cell culture

Metastatic tumor tissue was obtained immediately after surgery. After mechanical disaggregation into approximately 1–2 mm^3^ pieces, cells were cultured in DMEM (Invitrogen, Karlsruhe, Germany) containing 10% fetal bovine serum (FBS) (Biochrom, Berlin, Germany), 2 mM glutamine and 1% penicillin/streptomycin (Pen/Strep;) (both Invitrogen). A375 was used as a comparable cell line and cultured under the same conditions. Incubation was carried out at 37 °C in a humidified atmosphere of 5% CO_2_. All cell cultures were periodically checked for mycoplasma by PCR.

### Colony-forming units (CFUs)

Three six well plates (Corning, NY) were seeded with 400 cells per well and incubated at 37 °C and 5% CO_2_, at different time points After the incubation, cells were fixed with 4% formaldehyde and stained with 1 mg/ml Crystal Violet (Merck, Darmstadt, Germany) for 10 min at room temperature. All cells were cultured for 30 days, and the number of colonies formed per well was quantified.

### Flow cytometry

Single-cell sorting was performed on a FACS Aria II (BD Bioscience, San Jose, CA) after doublet discrimination and using the single cell sorting function. In total, five 96 plates were sorted of 5 × 10^5^cells/ml (passage 55) was sorted, Propidium iodide was used to exclude dead cells. 1 × 10^6^ cells were stained with CD271 (nerve growth factor receptor) Alexa Fluor 405, CD34 FITC, CD44 PE (all from BD Bioscience), with matched fluorochrome-conjugated isotype controls. Flow cytometry analysis was performed using a FACS LSR II System (BD Bioscience), and data were acquired by FACSDiva software (BD Bioscience).

### xCELLigence

Proliferation and migration assay was performed by using the real-time xCELLigence system. Respectively 5 × 10^3^ and 1 × 10^4^ MUG-Mel1, D5, C8, and optional A375 cells were seeded in E-Plate™ (ACEA Biosciences) and measured up to 92 h with the xCELLigence system according to the instructions in the user’s manual. For migration assay 3 × 10^4^ cells/well were used. A chemotactic signal for movement was provided by inoculating the cells in serum free medium in the upper chamber and by supplying 10% FBS in the lower chamber. Cell density measurements were performed in quadruplicate and measured every 20 min. Data acquisition and analyses were determined with the RTCA 2.0 software (OLS). All experiments were done in biological triplicates.

### Immunohistochemistry

Available tumor tissues and derived cell lines were stained with antibodies against Melan A, S-100 protein, tyrosinase, HMB 45 (all antibodies from Dako, Carpinteria, CA). The Envision Plus detection system (Dako) for all antibodies was used. Appropriate positive and negative controls were included.

### Cell line identification Power Plex^®^ 16 system

DNA from tumor tissue and cells was prepared using the QIAamp DNA Mini kit (Qiagen, Hilden, Germany) in accordance with the manufacturer’s protocol. After normalizing the DNA, 1 μl of each sample was amplified using the Power Plex® 16 System (Promega, Madison, WI) in a 10 μl reaction. One μl of the product was mixed with Hi-Di formamide (Applied Biosystems Inc., US) and Internal Lane Standard (ILS600), denatured and fractionated on an ABI 3730 Genetic Analyzer (Applied Biosystems Inc.). The resulting data were processed and evaluated using ABI Genemapper 4.0^41^.

### Affymetrix SNP6.0

Affymetrix GeneChip Human Mapping SNP 6.0 array was performed as described in the Genome-Wide Human SNP Nsp/Sty 6.0 User Guide (Affymetrix Inc., Santa Clara, CA). SNP 6.0 data were imported and normalized using the Genotyping Console 4.0 program default settings. Sample passing QC criteria were subsequently genotyped using the Birdseed (v2) algorithm. We used 60 raw HapMap data generated with the Affymetrix Genome-Wide Human SNP Array 6.0 as a reference. Data were obtained from the Affymetrix web site and used for normalization. For visualization of copy number state and loss of heterozygosity (LOH), Choromosome Analysis Suite 1.1 software was used^42^.

### Low coverage whole genome sequencing for copy number profiling

Genome wide copy number aberrations were established using low-coverage whole genome sequencing. Shotgun libraries were prepared using the TruSeq DNA LT Sample preparation Kit (Illumina, San Diego, CA) with slight modifications according to the manufacturer’s protocol. After concentrating the volume to 50 µl end repair, A-tailing and adapter ligation were performed following the manufacturer’s instructions. For selective amplification of the library fragments that have adapter molecules on both ends we used 15 PCR cycles. Libraries were pooled equimolarily and sequenced on an Illumina MiSeq in a 150 bp single read run. On the completion of the run data were base called demultiplexed on the instrument (provided as Illumina FASTQ 1.8 files, Phred + 33 encoding), and FASTQ format files in Illumina 1.8 format were used for downstream analysis. Copy number analysis was performed as previously described, for each segment, we calculated z-scores by comparing GC-corrected read counts for samples and controls^43^.

### Mutation Analysis using AmpliSeq

Genomic DNA of primary tumor, MUG Mel1 and clones was isolated on a Maxwell, MDxResearch System (Promega). Highly multiplexed PCR was used to generate amplicon libraries covering the coding region of 50 genes commonly implicated in cancer (Cancer Hotspot Panel v2, Cat.Nr. 4475346; Thermo Fisher Scientific, Waltham, MA). All analyses were performed in duplicates. Libraries were prepared using the Ion AmpliSeq Library Kit 2.0 and sequencing was performed on an Ion Proton Sequencer (Thermo Fisher Scientific). Emulsion PCR and sequencing runs were performed with the appropriate kits (Ion One Touch Template Kit version 2 and Ion Proton 200 Sequencing Kit; Thermo Fisher Scientific) using Ion PI chips. Sequencing length was set to 520 flows and yielded reads ranging from 70 to 150 bp, consistent with the expected amplicon size range. Initial data analysis was performed using the Ion Torrent Suite Software version 4.1 Plug-ins (Thermo Fisher Scientific)^44^.

### Cytogenetics, COBRA FISH

Cells were harvested using the chemically induced chromosome condensation technique^45^. Slides with metaphase chromosomes were hybridized using a multicolor FISH approach, known as COBRA-FISH. The 43-color FISH staining of every chromosome arm in a different color combination, digital imaging and analysis were performed as previously described^46^. Hybridizations with individual libraries labeled with single fluorochromes were used to confirm the detected rearrangements. Chromosomal breakpoints were assigned using inverted images counterstained with 4′,6-diamidino-2-phenylindole (DAPI; Downers Grove, IL) together with the information derived from the short- and long-arm specific hybridization during COBRA-FISH. Karyotypes were described according to ISCN 2009^47^.

### Generation of stable fluorescent MUG-Mel1 and derived clones

Plasmids were generated through In-Fusion cloning (Takara bioscience, Saint-Germain-en-Laye, France) inserting a lox-dsRED-stop-lox-eGFP cassette (derived from Addgene plasmid 32702) or eGFP-P2A-T2A (pUltra MCS, derived from Addgene plasmid #24129) into the lentiviral backbone pLenti- V6.3 (Thermo Fisher Scientific, Landsmeer, the Netherlands). Lentiviral particles were generated by transfection of HEK293T cells using 6 µg transgene encoding plasmid, supplemented with 2 µg PAX2 and 1ug of pMDG.2 virulence and packaging plasmids complexed with 34 µl of LipoD293t (Sino biologicals, Beijing, China) as modified from Campeau *et al*.^48^. MUG-Mel1 and derived clone C8 were transduced with lentiviral eGFP-P2A-T2A-Blas (pLenti-v6.3 Ultra, Addgene: plasmid #106172) and clone D5 was transduced with lentiviral lox-dsRED-stop-lox-eGFP-blas (Addgene: plasmid #106171) and subsequently selected for blasticidin (Thermo Fisher scientific) resistance through titration (1–10 µg/ml in 2 log dilution series) (Suppl. Figs 3–4).

### Engraftment of MUG-Mel1 cancer cells into zebrafish larvae

All zebrafish were raised and maintained in concordance with local animal welfare regulations in line with standard protocols (www.ZFIN.org). Zebrafish engraftments were performed as previously described in Chen *et al*.^49^.

Engraftment and disease progression was tracked using a Leica stereo fluorescent microscope (MZ16FA, Leica microsystems, Amsterdam, the Netherlands). To track the overall engraftment efficiency of the maternal line MUG-Mel1-tdTOM we used fli:GFP transgenic zebrafish, enabling the visualization the engrafted cancer cells in relation to both blood and lymphatic vessels^50^. Subsequently a competition assay was performed for both derived clones C8 and D5 by injection of mixed cells (1:1 ratio) labeled in green and red respectively. These cells were intravenously engrafted into ABTL (wild type) zebrafish. Representative embryos were selected and confocal images of the whole embryo were acquired using a Leica TCS SPE confocal microscope using a 5x fluotar dry objective (0.12 N.A). Images were subsequently stitched using Fiji^51^.

### Chemical fixation for electron microscopy

MUG-Mel1, C8, and D5 were grown on an Aclar film (Gröpl, Tulln, Austria), fixed in 2.5% (wt/vol) glutaraldehyde and 2% (wt/vol) paraformaldehyde in 0.1 M phosphate buffer, pH 7.4, for 2 h, postfixed in 2% (wt/vol) osmium tetroxide for 2 h at room temperature, dehydrated in graded series of ethanol and embedded in TAAB (Agar Scientific, Essex, GB) epoxy resin. Ultrathin sections (70 nm thick) were cut with a UC 7 Ultramicrotome (Leica Microsystems, Vienna, Austria) and stained with lead citrate for 5 min and with uranyl acetate (UAc) for 15 min. Images were taken using a Tecnai G2 20 transmission electron microscope (FEI, Eindhoven, Netherlands) with a Gatan ultrascan 1000 charge coupled device (CCD) camera (temperature −20 °C; acquisition software Digital Micrograph; Gatan, Munich, Germany). Acceleration voltage was 120 kV^52^.

### Analysis of lipids

Lipids were extracted by a previously published methyl-tert-butyl ether protocol^53^ and an aliquot of the lipid extract was resuspended in 100 µl isopropanol:chloroform:methanol (90:5:5 v/v/v). Fourfold experiments of MUG-Mell 1, C8 and D5 were carried out. Data were acquired in data dependent acquisition mode by an LTQ Orbitrap Velos Pro instrument (Thermo Scientific) coupled to an Accela UHPLC (Thermo Scientific) according to previously published protocols^54,55^ with 100.000 mass resolution at m/z 400. Data were analyzed by Lipid Data Analyzer (LDA), a custom developed lipidomics software package^56^.

### Tumorigenicity study

8 × 10^6^ MUG-Mel1/200 µl serum free medium was injected into ten six week old NU-Foxn1-nu female mice (Charles River Laboratories, Sulyfeld, Germany). Six week old female/male NOD/SCID/IL-2rγnull (NSG-) mice (Charles River Laboratories, Sulzfeld, Germany) were xenotransplanted with MUG-Mel1, MUG-Mel1 CD271+/CD271-, clone C8 and clone D5. Cell lines were suspended in 0.2 ml of serum-free medium and subcutaneously inoculated into the right flank of 10 mice. In accordance with a protocol approved by the committee for institutional animal care and use at the Austrian Federal Ministry of Science and Research (BMWF) (vote 66.010/0160-II/3b/2012), animal work was carefully carried out. Organotrophic injection: fixation of mice into a stereotactic frame; opening of skin covering the cranium with a scissor opening of the cranium with 16-gauge needle 3 mm lateral from median layer; insertion of a 10 µl syringe through this opening. Injection of tumor cells in a depth of 3 mm (1 µl/ min); total cell volume: 2 µl Removal of syringe in two steps: each 1,5 mm/min Wound closure with fibrin glue (Histoacryl/Braun) cell number: 2 × 10^4^/mouse mouse strain: NMRI:nu/nu, female (Janvier).

### Reverse transcription quantitative real-time -PCR (RT-qPCR)

RT-qPCR was performed in order to determine the relative expression of the stemness markers SOX-2, c-Myc, and E-cadherin. Total RNA was isolated with RNeasy Mini Kit (Qiagen) according to the manufacturer’s recommended protocol. RT-qPCR reactions were performed in triplicate using the Platinum SYBR Green Super Mix with ROX (Invitrogen) on AB7900HT (Applied Biosystems Inc.). The reference genes glyceraldehyde 3-phosphate dehydrogenase (*GAPDH*), β-actin (*ACTB*), and hypoxanthine phosphoribosyltransferase (*HPRT1*) were used for normalization and to demonstrate their stable expression in different tissues^31^. The following primers were used for RT-qPCR: QuantiTect primer assays (Qiagen) for c-Myc (ID QT00062069) and SOX-2 (ID QT00237601). The expression level (CT) of the target gene was normalized to the reference genes (*GAPDH, ACTB*, and *HPRT1*) (ΔCt) and the ΔCt of the test sample was then normalized to the ΔCt of the controls (ΔΔCt). Finally, the expression ratio was calculated with the 2−ΔΔCt-method^41,57^.

### Peptid uptake R-DIM-P-LF11-322 peptide

Cells were collected, re-suspended in media and diluted to a concentration of 10^6^ cells/ml. Aliquots of 10^5^ cells/100 µl media were incubated with different amounts of peptide (0–80 µM) for 8 h at 37 °C and 5% CO_2_. Propidium iodide (PI) (2 µl/10^5^cells of 50 µg/ml, Molecular Probes, Eugene, US) was added and cells incubated for 5 min at room temperature in the dark. Excitation and, emission wavelengths were 536 and 617 nm, respectively. Cytotoxicity was calculated from the percentage of PI positive cells in media alone (P_0_) and in the presence of peptide (P_X_). Triton-X-100 was used to determine 100% of PI positive cells (P100). %PI –uptake = 100 × (P_X_ − P_0_)/(P_100_ − P_0_).

### Ethics statement

All methods were carried out in accordance with relevant guidelines and regulations.

## Supplementary information

Supplementary data

## Data Availability

All data generated or analyzed during this study are included in this published article (and its Supplementary Information files).
