# Supplementary material for: Human melanoma brain metastases cell line MUG-Mel1, isolated clones and their detailed characterization
Source: Sci Rep. 2019 Mar 11;9:4096. doi: 10.1038/s41598-019-40570-1 (PMC6411871; doi:10.1038/s41598-019-40570-1)
Supplement: Supplementary file 1 — Supplementary data [file 41598_2019_40570_MOESM1_ESM.docx]

**Human melanoma brain metastases cell line MUG-Mel1, isolated clones and their detailed characterization**

Ellen Heitzer^1*^, Arwin Groenewoud^2*^, Katharina Meditz^3^, Birgit Lohberger^4^, Bernadette Liegl-Atzwanger^5^, Andreas Prokesch^6,7^, Karl Kashofer^5^, Diana Behrens^8^, Johannes Haybaeck^5,9^, Dagmar Kolb-Lenz^6,10^, Harald Koefeler^10^, Sabrina Riedl^11^, Helmut Schaider^12^, Carina Fischer^13^, B. Ewa Snaar-Jagalska^2^, Danielle de’Jong^14^, Karoly Szuhai^14^, Dagmar Zweytick^11**^ and Beate Rinner^3**^

^1^ Institute of Human Genetics, Medical University of Graz, Austria

^2^Gorlaeus Laboratories, Institute Biology Leiden, The Netherlands.

^3^ Division of Biomedical Research, Medical University of Graz, Austria

^4^ Department of Orthopedic Surgery, Medical University of Graz, Austria

^5^Institute of Pathology, Medical University of Graz, Austria

^6^ Gottfried Schatz Research Center for Cell Signaling, Metabolism & Aging, Medical University of Graz, Austria;

^7^BioTechMed-Graz, Graz, Austria

^8^EPO - Experimental Pharmacology and Oncology GmbH, Berlin, Germany.

^9^Department of Pathology, Medical Faculty, Otto von Guericke University Magdeburg, Germany

^10^Center for Medical Research, Medical University of Graz, Austria

^11^Institute of Molecular Biosciences, Biophysics Division, University of Graz, Austria

^12^Dermatology Research Centre, Translational Research Institute, School of Medicine, The University of Queensland, Brisbane, Queensland, Australia

^13^Red Cross Transfusion Service for Upper Austria, Austria.

^14^Department of Cell and Chemical Biology, LUMC, Leiden, The Netherlands

***first authors** contributed equally: Ellen Heitzer^1^ and Arwin Groenewoud^2^

****corresponding authors** contributed equally: Beate Rinner^3^ and Dagmar Zweytick^11^

Beate Rinner, Prof. PD PhD MSc

Division of Biomedical Research, Medical University of Graz, Roseggerweg 48, 8010 Graz, Austria; Phone: +43 316 385 73524; Email: [beate.rinner@medunigraz.at](mailto:beate.rinner@medunigraz.at)

Dagmar Zweytick, PhD DI

Institute of Molecular Biosciences, Biophysics Division, University of Graz, Humboldtstraße 50, 8010 Graz, Austria. Phone: +43 316 380 9897,

Email: [dagmar.zweytick@uni-graz.at](mailto:dagmar.zweytick@uni-graz.at)

To prove the common origin of the tumor samples, the parental cell line and the two subclones STR profiling was performed. All analyzed samples showed the same tandem repeat (STR) profile at the markers D3S1358, TH01, D18S51, D5S818, D13S317, D7S820, D16S539, CSF1PO, Penta D, vWA, D8S1179, TPOX, Amelogenin and FGA confirming that MUG-Mel1, C8, and D5 emanated from the same patient (Suppl Table 1).

**Suppl Table 1:** STR Profile

| STR-Locus | MUG-Mel1 Tissue | MUG-Mel1  p9 | MUG-Mel1 p97 | MUG-Mel1 C8 p63 | MUG-Mel1 D5 p63 |
| --- | --- | --- | --- | --- | --- |
|  |  |  |  |  |  |
| D3S1358 | 17, 18 | 17, 18 | 17, 18 | 17, 18 | 17, 18 |
| TH01 | 9, 3 | 9, 3 | 9, 3 | 9, 3 | 9, 3 |
| D21S11 | 31, 31.2 | 31, 31.2 | 31, 31.2 | 31, 31.2 | 31, 31.2 |
| D18S51 | 13, 14 | 13, 14 | 13, 14 | 13, 14 | 13, 14 |
| Penta E | 5, 12 | 5, 12 | 5, 12 | 5, 12 | 5, 12 |
| D5S818 | 12 | 12 | 12 | 12 | 12 |
| D13S317 | 11, 12 | 11, 12 | 11, 12 | 11, 12 | 11, 12 |
| D7S820 | 8, 11 | 8, 11 | 8, 11 | 8, 11 | 8, 11 |
| D16S539 | 11 | 11 | 11 | 11 | 11 |
| CSF1PO | 10, 11 | 10, 11 | 10, 11 | 10, 11 | 10, 11 |
| Penta D | 9, 10 | 9, 10 | 9, 10 | 9, 10 | 9, 10 |
| Amelogenin | x, y | x, y | x, y | x, y | x, y |
| vWA | 16 | 16 | 16 | 16 | 16 |
| D8S1179 | 13, 14 | 13, 14 | 13, 14 | 13, 14 | 13, 14 |
| TPOX | 8 | 8 | 8 | 8 | 8 |
| FGA | 21, 24 | 21, 24 | 21, 24 | 21, 24 | 21, 24 |

**Suppl Table 2:** Primary Tumor Variants


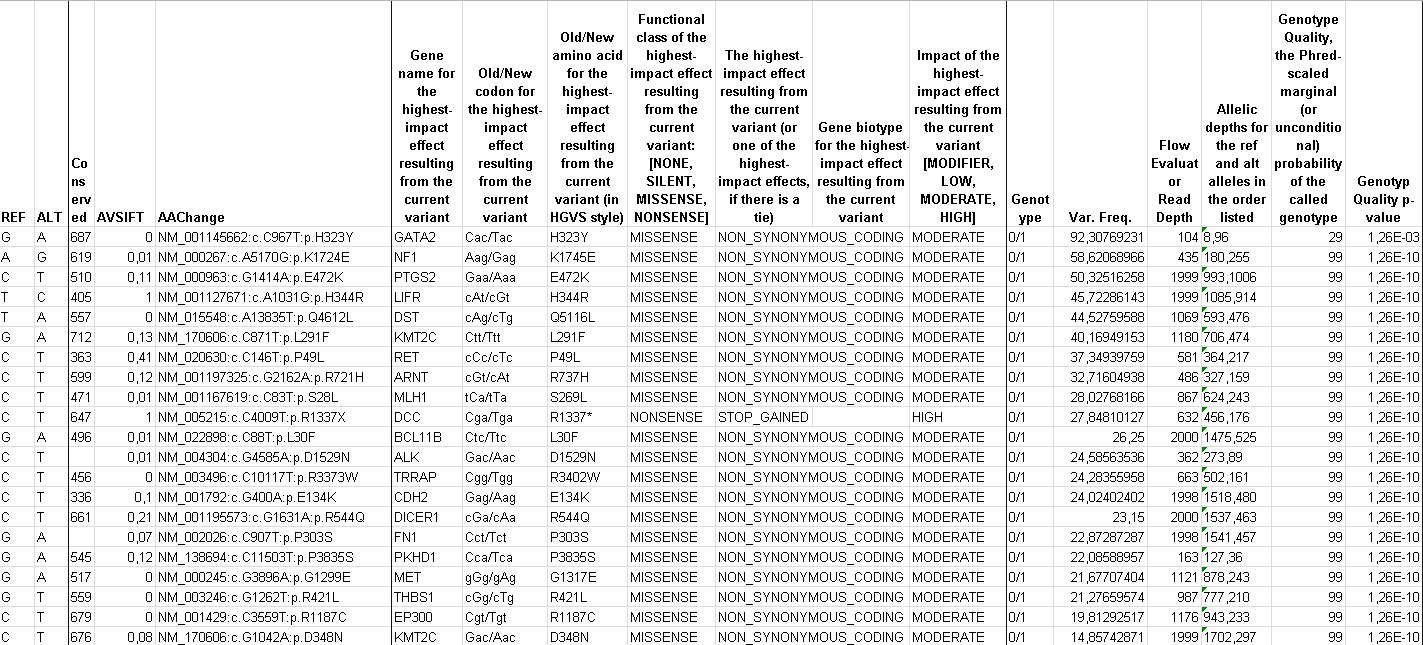


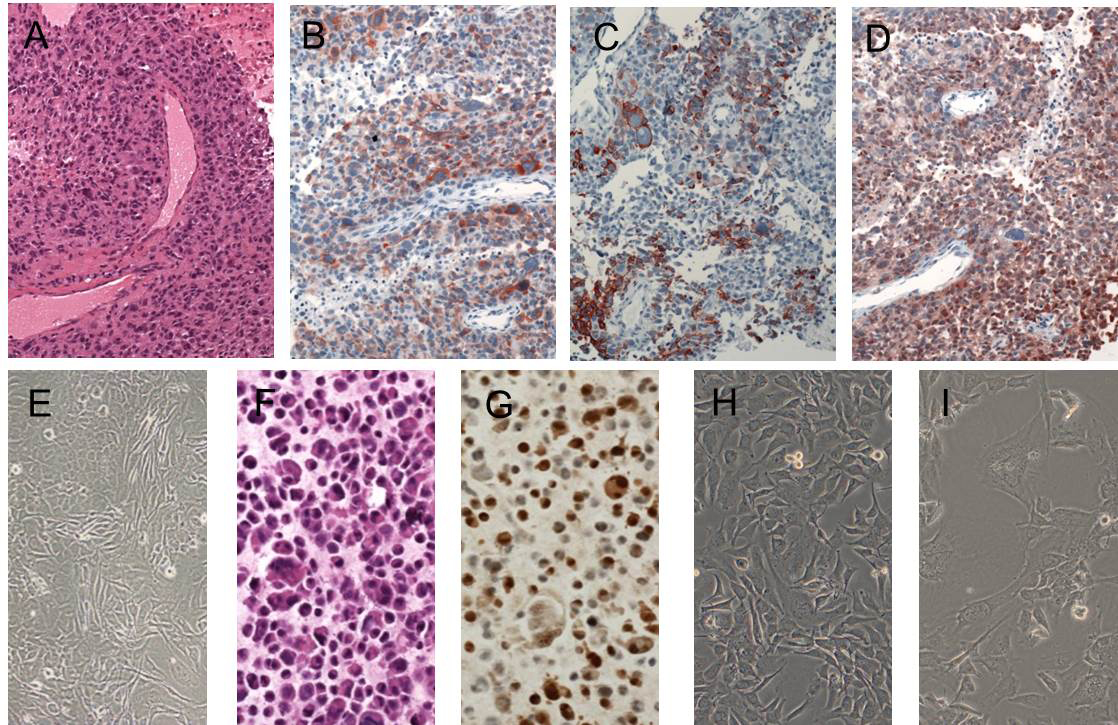


**Suppl Fig. 1:** Morphological and immunohistochemical characterization, A-D.) Melanoma brain tissue: HE staining (A), partly positive Melan A staining (B), partly positive HMB45 staining (C), strong positive S100 staining (D), E-F Morphological features of MUG Mel1 cell line and resulting clones; MUG-Mel1 cell line wildtype (E), HE staining MUG-Mel1 WT (F), S100 staining MUG-Mel1 WT (G), spindle shaped growth MUG-Mel1 clone C8 (F), round and flat growth MUG-Mel1 clone D5 (G), x20 magnification.

**
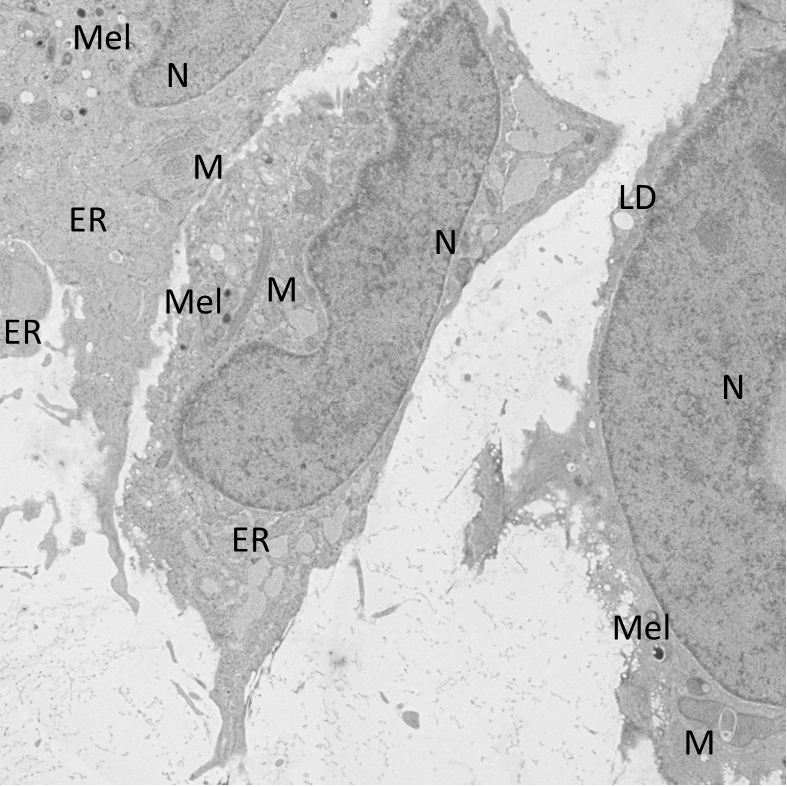
**

**Suppl Fig. 2:** Electron microscopy overview of MUG Mel 1 cells (stitched image).

High pressure freezing fixation was used to demonstrate the ultrastructure of MUG Mel1 cells. Cells showed a prominent nucleus (N), dilated endoplasmatic reticulum (ER), melanosomes (Mel), (elongated) mitochondria (M), Lysosomes (L) and Lipid droplet (LD**).**

**
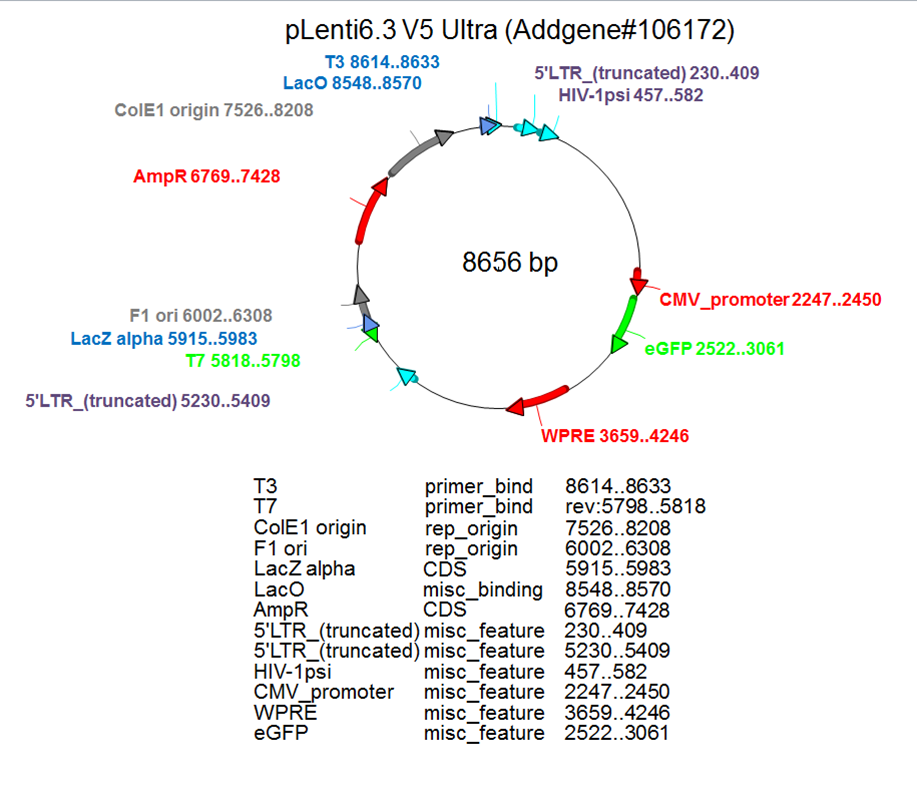
**

**Suppl Figure 3:** pLenti6.3 V5 Ultra


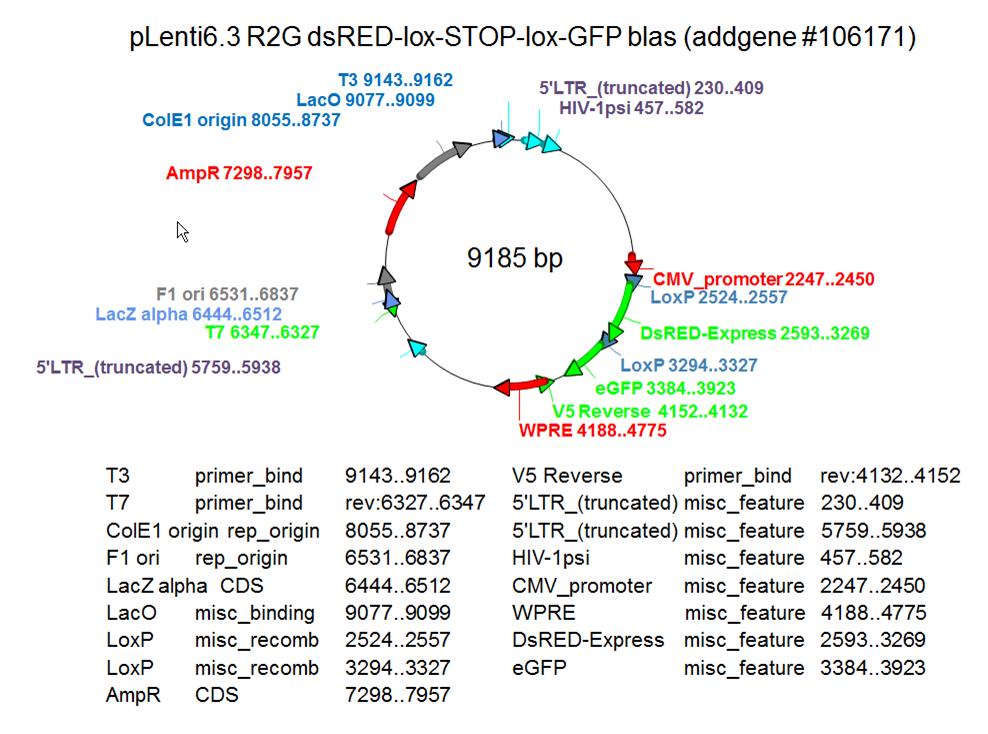


**Suppl Figure 4:** pLenti6.3 R2G dsRED-lox-STOP-lox-GFP blas
